# Cognitive Sensory Motor Training Therapy in Stroke Patients: A Systematic Review

**DOI:** 10.31083/RN50584

**Published:** 2026-06-30

**Authors:** Mirari Ochandorena-Acha, Sandra Rierola-Fochs, Jose Antonio Merchán-Baeza, Manuel González-Sánchez, David Pérez-Cruzado

**Affiliations:** ^1^Technology, Inclusion and Health Research Group (TIS), University of Vic-Central University of Catalonia (UVic-UCC), 08500 Vic, Spain; ^2^Department of Psychiatry and Physiotherapy, Institute of Biomedical Research of Malaga (IBIMA), University of Malaga, 29071 Malaga, Spain

**Keywords:** Perfetti’s method, stroke, rehabilitation, upper-limb, recovery, function, neurorehabilitation, systematic review

## Abstract

**Background:**

Stroke patients’ sensorimotor function is often affected, leading them to experience limitations in daily living activities. Cognitive sensorimotor training therapy, or Perfetti’s method, is designed to recuperate both motor and sensory aspects through activating cognitive processes. The purpose of the study was to determine the effectiveness of cognitive sensorimotor training therapy compared with other interventions in enabling the recovery of upper-limb function after a stroke.

**Methods:**

A systematic literature review was conducted on Perfetti’s method from the first available publication to January 2026, consulting the PubMed, Web of Science, and Scopus databases. Two independent authors performed the search. The Physiotherapy Evidence Database (PEDro) scale was used to assess the internal validity.

**Results:**

Four studies with a variety of methodological design were included. Eight outcome variables related to upper limb’s recovery were analyzed. Upper-limb function was the most studied outcome, but only one study showed statistically significant differences between groups. Overall, participants undergoing Perfetti’s method tended to show greater post-intervention improvements compared with control interventions; however, between-group differences were generally not statistically significant.

**Conclusions:**

The included studies show heterogeneity regarding duration and intensity, among others, and it is difficult to draw robust conclusions. However, the results indicate some clinical benefits of Perfetti’s method, although further well-designed studies are needed.

## 1. Introduction

Since 2000, stroke has been the second leading cause of death in adults, responsible for approximately 11% of total deaths. This condition also stands as one of the primary contributors to acquired disabilities in adults [1]. About 80% of survivors have disabilities that generally affect the sensitivity and movement of the face, the arm, and the leg on one side of the body due to neuronal cell death [2,3,4]. In fact, stroke recovery is heterogeneous in terms of outcomes, with an estimated 25% to 74% of survivors worldwide requiring help or becoming dependent on caregivers [5].

After a stroke, patients’ predominant tendency is to use the unaffected limb and not consider the paretic limb, thus developing learned non-use [6]. Moreover, mood and cognitive ability can be negatively affected, further decreasing functional abilities and motor impairment of the upper limb itself [3,4,7,8]. Consequently, decreased functionality leads to limitations in the performance of activities of daily living (ADL), loss of significant activities, and reduced social participation, affecting the person’s well-being and quality of life [3,4,7,9].

Interdisciplinary complex rehabilitation interventions, such as physiotherapy and occupational therapy, are aimed at restoring and maintaining ADL and usually start within the first few days and often continue into the chronic phase post-stroke [2,4]. During the motor rehabilitation, intact sensations are considered a critical component, as somatosensory inputs intervene in motor control and motor skills acquisition [3,8]. Integrating bodily and environmental information about the different senses is fundamental for optimal motor performance in daily activities [10]. Therefore, sensory input plays an essential role in motor recovery after a stroke [10]. Sensitivity allows us to detect and discriminate objects and textures, know where our body is in space, and accurately perceive and discriminate sensations such as pain, temperature, pressure, and vibration [3]. However, sensory deficits might negatively influence performance and motor recovery, especially in terms of hand function, where motor and sensory deficits are closely connected [11,12]. Consequently, somatosensory discrimination tasks are used during the rehabilitation of stroke patients. Since active movements are hampered in these people, passive somatosensory discrimination exercises are applied to stimulate the somatosensory system and improve motor recovery [13,14].

Cognitive sensorimotor training therapy, or Perfetti’s method, is a neurocognitive rehabilitation approach that aims to improve the organization and execution of voluntary movement through cognitively guided therapeutic exercises [15]. These exercises are considered “cognitive” because they require the individual to interact with the environment, process sensory information, classify and store experiences, and use them to guide future actions [15,16]. From this perspective, motor recovery is not solely dependent on muscular activation, but is strongly influenced by cognitive processes that determine the quality and organization of movement [16] The neurocognitive theory of rehabilitation underlying Perfetti’s method proposes that motor recovery depends on the type and level of cognitive processes activated during rehabilitation, including attention, perception, memory, language, and mental representation [15]. This view is supported by contemporary neurophysiological evidence, which highlights the distributed nature of sensorimotor processing. In particular, cortical regions such as the parietal and premotor areas are involved not only in motor execution but also in the integration of sensory information stored in memory to support motor planning, decision-making, and action evaluation [15]. Furthermore, research on mirror neurons and parietofrontal networks suggests a shared neural substrate for perception and action, reinforcing the concept of an integrated sensorimotor-cognitive system involved in motor organization [15]. Clinically, this perspective supports rehabilitation strategies that emphasize perceptual-cognitive engagement through guided sensory-motor interaction and therapist-mediated feedback [16].

Originally developed by Carlo Perfetti in the 1970s, the method emerged from neurorehabilitation studies of patients with central nervous system damage and has since been framed within cognitive rehabilitation theory as an approach aimed at improving motor recovery after neurological injury. It is considered a holistic rehabilitation approach that integrates sensory and motor training through cognitive mediation. The method is based on the idea that movement is the result of complex interactions among multiple cognitive functions [6,15], requiring patients to solve perceptual-cognitive problems through bodily experience while focusing attention on movement and sensory feedback [16]. It also includes principles such as guided passive mobilization, active exploration, and therapist-mediated cognitive engagement [6,15]. Despite its theoretical rationale and clinical use in some rehabilitation settings, the effectiveness of Perfetti’s method remains controversial. The current evidence is limited and heterogeneous, characterized by small sample sizes, variability in intervention protocols, and a scarcity of high-quality randomized controlled trials. As a result, its superiority over conventional physiotherapy or occupational therapy has not been consistently demonstrated, and there is still no clear consensus regarding its clinical effectiveness in stroke rehabilitation. Accordingly, this systematic review aims to determine the effectiveness of cognitive sensorimotor training therapy or Perfetti’s method, compared to other intervention methods in enabling the recovery of upper-limb function in people who have suffered a stroke, both in the acute and chronic stage.

## 2. Material and Methods

### 2.1 Study Design

This systematic review was carried out following the Cochrane Handbook for Systematic Reviews of Interventions [17] and the Preferred Reporting Items for Systematic Reviews and Meta-Analysis (PRISMA) guide [18]. The PRISMA checklist is available in the **Supplementary Material**.

### 2.2 Search Strategy and Selection Criteria

The authors created the research question following the PICOS model (Participants, Intervention, Comparisons, Outcome, and Study design) [19]: ‘How effective is Perfetti’s method or cognitive sensorimotor training therapy for improving functions of the upper limb in people who have suffered a stroke?’. Eligible study design (S) included randomized controlled trials, and pilot studies evaluating the effects of Perfetti’s method were considered eligible. Given the limited number of studies available on this topic, a broad range of study designs was included to provide a comprehensive overview of the existing evidence. It was considered appropriate for mapping the extent of the literature.

A literature review was conducted from the first available publication to January 2026 in the PubMed (https://pubmed.gov), Web of Science (https://www.webofscience.com/), and Scopus databases (https://www.scopus.com/). No specific search of grey literature sources or clinical trial registries was performed. The search terms combined controlled vocabulary (MeSH terms in PubMed) and free-text terms. The following keywords were used: (‘recovery of function’ OR ‘neurological rehabilitation’ OR ‘physical education and training’ OR neurological) AND (‘cognitive therapy’ OR ‘sensory deprivation’ OR Perfetti OR ‘cognitive sensory motor training’ OR ‘cognitive therapeutic exercises’ OR ‘cognitive exercise therapy’ OR neurocognitive) AND stroke. Two authors (DPC and JAMB) independently screened the studies identified in the literature review. First, they evaluated the titles and the abstracts of the studies to assess their eligibility. In the second phase, the full texts of the remaining articles were assessed. Disagreements in selecting the studies between the two authors were resolved in consensus by consulting the full text again. When the disagreements persisted, a third reviewer (MGS) assessed the eligibility of the research.

The inclusion criteria were clinical trials and case studies written in English or Spanish and with no limit on the publication date. Included studies had to implement cognitive sensorimotor training therapy, or Perfetti’s method, as an upper-limb intervention in people who had suffered a stroke in the acute or chronic stage. All those publications that did not appear in peer-reviewed journals were excluded.

In order to minimize the risk of bias, an internal validity analysis of the included articles was conducted using the Physiotherapy Evidence Database (PEDro) scale [20].

### 2.3 Internal Validity Analysis

The internal validity analysis was carried out using the PEDro scale, which has excellent reliability (intraclass correlation coefficient (ICC) = 0.68; 95% confidence interval = 0.57–0.76) [21]. The PEDro scale consists of 10 items that evaluate the selection of criteria, the allocation and randomization of the sample, blinding of membership in both groups, baseline characteristics of the groups, blinding of the subjects, therapists, and evaluators, data collection in at least 85% of participants, presentation of data from all participants, comparison between both groups and measures of variability.

A cutoff score of ≥6 was applied to identify studies with at least moderate-to-high methodological quality. For randomized controlled trials, this threshold corresponds to “good” methodological quality according to the PEDro scale. Although the PEDro scale was originally developed for randomized controlled trials, it was applied to all included studies to ensure consistency in methodological quality assessment. However, its applicability to pilot studies may be limited, and the resulting scores should therefore be interpreted with caution.

Two authors (DPC and JAMB) independently performed the assessment (Table 1, Ref. [16,22,23,24]). Discrepancies were resolved by discussion, and the involvement of a third reviewer (MGS) was not required. Rather than being used as strict exclusion criteria, the quality scores were considered to aid the interpretation and weighting of the evidence within the synthesis of results.

**Table 1.** **Internal validity analysis**.

Author(s)	1	2	3	4	5	6	7	8	9	10	Total
Chanubol et al. 2012 [22]	●	●	●	-	-	●	●	●	●	●	8
Lee et al., 2015 [16]	●	●	●	-	-	-	●	●	●	●	7
Sallés et al. 2017 [24]	●	●	●	-	-	●	●	-	●	●	7
Metzger et al., 2014 [23]	●	●	●	-	-	●	-	●	●	●	7

●: Yes; -: No.

### 2.4 Extracted Variables and Statistical Analysis

Two authors (SRF and MOA), working independently and using a template, extracted the following information about all the studies (Table 2, Ref. [16,22,23,24]): source (including authors and year of publication), trial design, the profile of trial participants (diagnosis), sample size, details of the intervention (type, session length, frequency, and intervention length) and type of outcome measure and measurement time. In turn, the data recorded for each variable, pre- and post-intervention, and the significance of intragroup and intergroup differences were extracted.

**Table 2.** **Characteristics of included studies**.

Author(s)	Study design	Participants’ profile	Study groups and number of participants	Details of the intervention				Outcomes and measurement			
				Type	Session length	Frequency	Intervention length		Pre	Post	Follow-up
Chanubol et al., 2012 [22]	RCT	Acute stroke	CG: n = 20	Occupational therapy	30 min/day	5 days/week	4 weeks	Upper limb function and activity level Gross manual dexterity Level of dependence	NR	NR	No follow-up
			IG: n = 20	Perfetti's method	30 min/day	5 days/week	4 weeks				
Lee et al., 2015 [16]	Pilot RCT	Chronic stroke	CG: n = 8	Range of motion exercise and task-oriented exercise	60 min/day	5 days/week	8 weeks	Motor recovery of upper-limb Manual function ADL function Quality of life	NR	NR	No follow-up
			IG: n = 8	Perfetti's method	60 min/day	5 days/week	8 weeks				
Metzger et al., 2014 [23]	Pilot Study	Neurological disorder	n = 5	Robot-assisted Perfetti's method	45 min/day	3–6 days/week	2 weeks	Motor recovery of upper-limb and hand function	NR	NR	No follow-up
Sallés et al., 2017 [24]	Pilot Study	Subacute Stroke	CG: n = 4	Conventional physical therapy	30 min/day	3 days/week	10 weeks	Upper limb functionality Upper limb motricity Sensory impairment	NR	NR*	NR
			IG: n = 4	Perfetti's method	30 min/day	3 days/week	10 weeks				

RCT, randomized controlled trial; ADL, activities of daily living; CG, control group; IG, intervention group; NR, not reported; * middle assessment.

Effect size and the 95% confidence intervals were calculated for all parameters observed (see Table 3, Ref. [16,22,23,24]). For interpretation, the Cohen definition of effect size was used (d = 0.2 small, d = 0.5 medium, d = 0.8 large) [25]. Given the substantial heterogeneity across studies in terms of design, populations, interventions, and outcome measures, these values should be interpreted descriptively as within-study estimates and not as pooled or directly comparable measures across studies.

**Table 3.** **Comparison of results between groups providing the magnitude of change**.

Author(s)	Outcomes		Pre-intervention Mean ± SD	Post-intervention Mean ± SD	Follow-up	Significant difference		Between group effect size (Cohen’s d)
						Inter group	Intra group	
Chanubol et al., 2012 [22]	Upper limb function and activity level (ARAT)		CG: 16.40 ± 17.96 IG: 12.45 ± 15.06	CG: 11.00 ± 9.07 IG: 15.40 ± 11.38	No Follow-up	○	CG: ○ IG: ○	0.22
	Gross manual dexterity (Box and Block)		CG: 7.44 ± 12.17 IG: 7.53 ± 11.80	CG: 8.25 ± 10.42 IG: 13.82 ± 12.02		○	CG: ● IG: ●	0.52
	Level of dependence (Extended Barthel index)		CG: 43.65 ± 9.98 IG: 41.90 ± 8.48	CG: 58.6 ± 6.47 IG: 57.05 ± 9.70		○	CG: ● IG: ●	1.66
Lee et al., 2015 [16]	Motor recovery of upper-limb (Fugl-Meyer)		CG: 50.50 ± 6.80 IG: 53.50 ± 3.20	CG: 58.70 ± 2.50 IG: 62.20 ± 1.30	No Follow-up	●	CG: ● IG: ●	3.56
	Upper limb function (Manual Function test)		CG: 24.30 ± 3.10 IG: 25.50 ± 3.80	CG: 25.20 ± 3.10 IG: 30.30 ± 1.40		●	CG: ● IG: ●	1.67
	ADL functions (Motor activity log)	Quality of movement	CG: 82.3 ± 22.5 IG: 86.5 ± 20.4	CG: 112.30 ± 23.10 IG: 121.80 ± 24.70		○	CG: ● IG: ●	0.40
		Amount of use	CG: 53.80 ± 40.90 IG: 52.70 ± 14.60	CG: 102.50 ± 34.90 IG: 116.50 ± 29.00		○	CG: ● IG: ●	2.77
	Quality of life (Stroke Impact Scale)		CG: 57.60 ± 14.50 IG: 55.40 ± 6.40	CG: 63.20 ± 12.50 IG: 76.70 ± 5.60		●	CG: ● IG: ●	3.54
Metzger et al., 2014 [23]	Motor recovery of upper-limb (Fugl-Meyer)		39.4 ± 18.13	NR	No Follow-up	NR	CG: NR IG: ●	NR
	Motor recovery of hand (Fugl-Meyer subscore hand function)		9.20 ± 4.83	NR		NR	NR	NR
Sallés et al., 2017 [24]	Upper limb function (Motor Evaluation Scale for Upper Extremity in Stroke Patients)	Arm+	GC: 18 (12, 29.5) IG: 32.5 (29, 35.5)	GC: 22 (11, 34.5) IG: 38 (36, 40)	NR	NR	NR	No possible to calculate
		Hand+	GC: 2 (0.5, 4.5) IG:8.5 (8, 12.5)	GC: 6.5 (0, 15.5) IG:17.5 (15, 18)	NR	NR	NR	
	Upper limb motricity (Motricity Index)+		GC: 39.5 (26.5, 54.5) IG: 67 (58.5, 73)	GC: 51 (32, 76) IG: 77 (77, 85)	NR	NR	NR	
	Sensory function (Revised Nottingham Sensory Assessment)+	Light touch	GC: 8 (6, 8) IG:4 (3.5, 5.5)	GC: 8 (6, 8) IG: 6 (6.5 (5, 8)	NR	NR	NR	
		Kinesthesia	GC: 8.5 (8, 10.5) IG: 10 (8.5, 10.5)	GC: 11 (10, 12) IG: 12 (11, 12)	NR	NR	NR	

Cohen’s d: Effect sizes represent between-group differences based on post-intervention mean and standard deviation values. ADL, activities of daily living; ARAT, The action research arm test; NR, not reported; +, median (minimum, maximum); ●, statistically significant difference; ○, no statistically significant difference.

### 2.5 Data Synthesis

Given the heterogeneity of the included studies in terms of study design, participant characteristics, intervention protocols, and outcome measures, a meta-analysis was not performed. Instead, a narrative synthesis approach was adopted to summarize and interpret the findings.

Results were organized according to the main outcome domains (upper-limb function, motor recovery, sensory function, level of dependence, and quality of life). Within each domain, findings were compared descriptively across studies, considering pre- and post-intervention changes, between-group differences, and statistical significance when reported.

Effect sizes (Cohen’s d) were calculated when possible to provide a standardized estimate of intervention effects. Specifically, effect sizes were derived from post-intervention mean and standard deviation values to estimate between-group differences between the intervention and control groups. However, these were interpreted cautiously due to methodological and clinical heterogeneity across studies and were not used to perform pooled comparisons.

## 3. Results

**Fig. 1.**
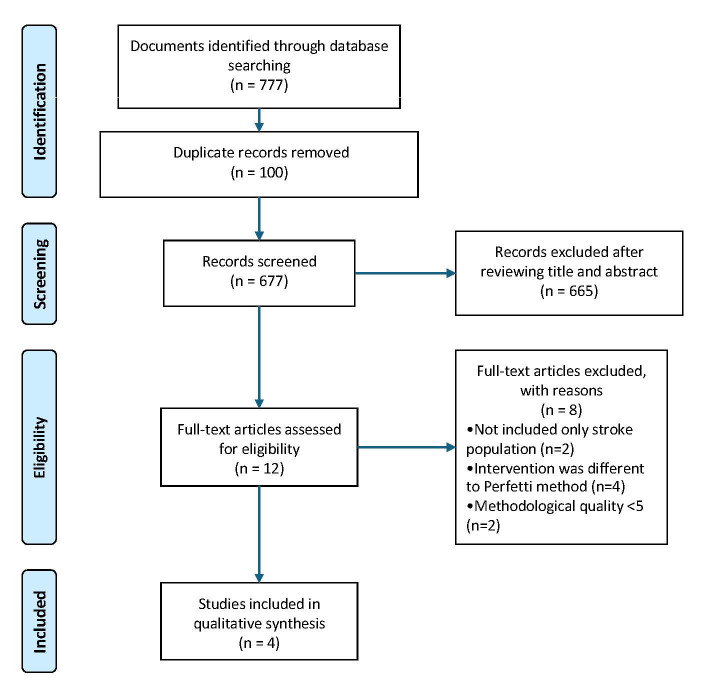
**Flow diagram**.

After establishing the search strategy and applying the inclusion and exclusion criteria, a search of the available scientific literature was carried out, obtaining a total of 777 articles, of which 100 were excluded after duplicates were removed. Of these 677 articles, 665 were eliminated after a review of the title and abstract due to not being related to the subject of study. These were primarily excluded due to not meeting the inclusion criteria, including irrelevant population (non-stroke), intervention not related to Perfetti’s method, outcomes not focused on upper-limb function, or study design not meeting eligibility criteria. Finally, the remaining 12 full-text articles were reviewed. Six articles were excluded because the population did not include only stroke participants, or the intervention was not based on Perfetti’s method. Thus, six articles were included in the internal validity analysis using the PEDro scale. Two of them obtained a score of ≤5 [26], so they were excluded in the present study (Fig. 1).

Table 1 shows the included articles’ assigned scores in the internal validity analysis carried out using the PEDro scale. In this analysis, four papers that had passed the previous filters were included, with a scores ranging 7 to 8 points. One of the included studies had a randomized controlled trial (RCT) design [22], and three were pilot studies [23,24,27]. Therefore, the level of evidence varies substantially across studies, with only one study providing high-level evidence, while the remaining studies represent lower levels of evidence and are more prone to bias.

This variability in study design and methodological quality may have influenced both the magnitude and consistency of the reported effects, potentially contributing to the observed heterogeneity in outcomes across studies. Therefore, the overall findings should be interpreted with caution, particularly regarding the strength and generalizability of the intervention effects.

Table 2 shows the characteristics of the studies included in the systematic review, such as study design, participants’ profile, sample size, intervention type in both groups and details of experimental intervention, and outcome measurements. The included studies’ participants were people with acute [22], subacute [27], and chronic stroke [24,28], and neurological disorder (without specifying the pathology) [23]. With regard to the upper limb, many outcomes were examined across the articles, such as upper-limb functionality, level of dependence, sensory function, upper-limb motricity, motor recovery, and gross motor dexterity. Finally, Perfetti’s method was compared with different types of therapies, such as conventional occupational therapy [22] or conventional physical therapy [27]. These variations across participant characteristics, intervention protocols, outcome measures, and study designs reflect substantial clinical and methodological heterogeneity across studies.

Table 3 summarizes the variables assessed in each study, the measurement instruments used, the results of intra- and between-group comparisons, and the corresponding levels of statistical significance. For each study, the effect sizes (Cohen’s d) were calculated based on post-intervention mean and standard deviation values to estimate between-group differences. Effect sizes are presented on a study-by-study basis and should be interpreted with caution; given the heterogeneity across studies, they represent within-study estimates and should not be directly compared or interpreted as pooled evidence. Instead, they should be considered as descriptive indicators within individual studies. It is important to note that only one study included follow-up of the sample after the intervention [27].

### 3.1 Upper-Limb Functionality

Four included studies analyzed the effect of the Perfetti's method compared to other interventions on upper-extremity function after stroke [22,24,27,28]. The Action Research Arm Test (ARAT) was used to assess the upper extremity’s function in two of the included studies [22,28]. The Motor Evaluation Scale for Upper Extremity in Stroke Patients [27] and the Manual Function Test [24] were also used to assess stroke participants’ upper extremity. The results were compared pre- and post-intervention in both groups and between groups. Some studies reported greater improvements in upper-limb function in the experimental group compared to the control group [22,27]. However, statistically significant between-group differences were only observed in the study conducted by Lee et al. (2015) [16], which reported large effect sizes [24]. The study by Chanubol et al. (2012) [22] observed an improvement in the upper extremity’s motor function after the intervention in the experimental group [22]. However, the differences between groups were not statistically significant, and the effect size was small (d = 0.22). It is important to note that the control group showed a decline in upper-limb function over time, which is not typically expected in acute stroke populations. This may have influenced the observed effect size and should be considered when interpreting the results. The other two studies [27,28] did not report these results.

### 3.2 Level of Dependence

Two included studies (50%) assessed the participants’ level of dependence through the extended Barthel Index scale [22] and the Motor Activity Log [24]. The results showed an improvement in the level of dependency between groups. The intra-group difference was statistically significant in the study by Chanubol et al. [22] with an effect size of d = 1.66 [22], but not inter-group difference either in Lee et al.’s research [16], although the effect size was large (d = 2.77) [24]. The results indicated a statistically significant improvement in both included studies within the control group and the experimental group, both in terms of movement quality and the amount of use.

### 3.3 Sensory Function

One (25%) of the included studies [27] assessed the sensitivity function through the revised Nottingham Sensory Assessment Scale. The abilities of soft touch and kinesthesia were assessed. The results showed a more statistically significant improvement in the experimental group’s touch than in that of the control group. The authors did not report whether the improvements were statistically significant, or whether the difference was statistically significant within groups. Moreover, kinesthesia improved in both the control and experimental groups, but it was not reported whether the improvement was statistically significant between or within groups. It should be noted that some statistical information was not reported in the original study (NR), which limits the interpretation of these findings.

### 3.4 Upper-Limb Motricity

One (25%) of the four included studies [27] assessed the upper limb’s motricity through the Motricity Index scale. The results showed a higher motricity of the upper extremity in the experimental group. However, it was not reported whether the difference was statistically significant in the control group. The authors displayed an improvement in both the experimental and post-intervention control groups, but the significance of this improvement was not reported. It should be noted that some statistical information was not reported in the original study (NR), which should be considered when interpreting these results.

### 3.5 Motor Recovery of the Upper Limb

Two (50%) of the included studies [23,24] assessed the upper extremity’s motor recovery using the Fugl-Meyer scale (FM). In Lee et al.’s study [16], the results showed a statistically significant improvement both within and between groups, with an effect size of d = 3.56 [24]. On the other hand, the research by Metzger displayed a statistically significant within-group improvement, but it was not reported whether there was an improvement between groups [23].

### 3.6 Quality of Life

One (25%) of the four included studies [24] assessed quality of life through the Stroke Impact Scale. The results showed a statistically significant improvement in the experimental group participants compared to the control group with an effect size of d = 3.54 and a statistically significant within-group improvement in both groups.

### 3.7 Gross Manual Dexterity

One (25%) of the included studies [22] assessed the gross manual dexterity through the Box and Block test. The authors displayed a statistically significant within-group improvement in both the control and experimental group after the intervention, and a lack of statistically significant improvement between groups with an effect size d = 0.52.

## 4. Discussion

The present systematic review aimed to analyze the effectiveness of Perfetti’s method or cognitive sensorimotor training therapy in recovering the upper-limb after a stroke. Overall, the included studies suggested a tendency toward greater post-intervention improvements in the intervention groups compared to control groups [22,27]. However, between-group differences were rarely statistically significant [22,23,27], and the interpretation of these findings is limited by the heterogeneity and the methodological quality of the included studies.

Upper-limb function was studied in four of the included studies [22,24,27,28]. The results showed greater differences between pre- and post-intervention measurements in the intervention group than in the control group, which underwent conventional physical [27] or occupational [22] therapy, and a range of motion and task-oriented exercises [24]. However, only two of the studies made intergroup comparisons [22,24]. These studies reported effect sizes ranging from 0.22 and 1.67 [22,24], with statistically significant differences between groups only being found in the research conducted by Lee et al. [16]. In this context, both studies included people who had suffered a stroke, but Lee et al.’s [16] participants were in the chronic phase and Chanubol et al.’s [22] were in the acute phase. This difference in clinical stage may partially explain the variability in findings, although other factors associated with chronic conditions, such as social, cognitive, physical, and lifestyle, may also influence post-stroke recovery trajectories [29]. Given the differences in study design and methodological rigor, these findings should be interpreted with caution.

Another factor that may help explain the differences observed between these studies is the intensity and duration of the cognitive sensorimotor training therapy. The study conducted by Lee et al. [16] applied 60 minutes per day for eight weeks [24], while the research carried out by Chanubol et al. [22] applied 30 minutes per day for four weeks [22]. Although differences in intervention intensity and duration may partly explain the variability in outcomes, the current evidence is insufficient to establish a clear dose–response relationship. Therefore, any interpretation regarding treatment dosage should be made with caution. When discussing the effectiveness of different rehabilitation therapies, it is important to consider that both timing and dosage may influence motor outcome, particularly in chronic stages of recovery [29,30]. However, the relationship between intervention dose and motor recovery remains unclear, as current evidence does not consistently support the hypothesis that higher doses lead to better outcomes [31]. Consequently, while intervention intensity may be a relevant factor, its specific contribution to recovery cannot be determined based on the available evidence [32].

In terms of upper-limb level of dependence, only two of the included studies evaluated this variable [22,24]. Both studies performed between-group comparisons and reported more favourable outcomes in the intervention group, with Cohen’s d values ranging from 1.66 to 2.77 [16,22]. However, these differences were not statistically significant compared to the control group. Similarly, the research conducted by Lee et al. [16] reported improvements in quality of life in participants who underwent Perfetti’s method [24]. Additionally, studies assessing sensory function [27], upper-limb motricity [27], and motor recovery [23,24] (predominantly pilot studies, including one pilot randomized controlled trial), generally showed more favourable outcomes in the intervention group. However, these findings were not consistently supported by statistically significant between-group differences and should therefore be interpreted with caution.

From a theoretical perspective, these findings may be explained by the sensory and cognitive components underlying Perfetti’s method. This approach is consistent with current theories of neural plasticity and cognitive rehabilitation, which emphasize the role of active engagement, sensory feedback, and cognitive processes in promoting motor recovery after stroke [33,34]. However, it is important to emphasize that these interpretations remain speculative, as the included studies did not directly assess the underlying neurocognitive mechanisms. Previous research has highlighted the association between cognitive function and upper-limb motor recovery, with initial cognitive status being a predictor of functional outcomes [35,36]. This supports the relevance of integrating cognitive processes into rehabilitation programs. In this context, improvements in upper-limb function may be associated with lower levels of dependency and better quality of life, although the direction of this relationship remains unclear [37,38]. Additionally, the observed improvements could be related to the sensory and cognitive demands of the intervention [39]. However, it is important to note that these mechanisms were not directly assessed in the included studies. Therefore, these interpretations should be considered as theoretical hypotheses rather than evidence derived from the present review.

More specifically, in terms of upper-limb sensitivity, this appears to be an essential aspect of Perfetti’s method [3]. The organization of movement requires the activation of several structures working in parallel to integrate sensory and motor information and transform it into motor actions [27]. Current knowledge on motor organization shows that sensory and motor information has a common neural substrate in the parietofrontal circuit, which allows the creation of a motor system that includes other more cognitive functions such as perception, imitation, understanding of gestures, and the intention of other people’s actions [40,41]. In fact, Perfetti’s method consists of providing the person with learning experiences and sensory stimuli enriched by the interaction with a therapist [27]. However, only one of the included studies directly assessed sensory function [27], which limits the ability to draw conclusions about the role of sensory mechanisms in the observed outcomes.

The studies included in this systematic review showed considerable clinical and methodological heterogeneity in terms of participant characteristics, outcome variables and assessment tools, intervention protocols, and study designs. This variability arises from differences in stroke phase (acute vs. chronic), baseline functional status, the specific implementation and dosage of Perfetti’s method, and the use of diverse outcome measures to assess upper-limb function. In addition, the inclusion of both randomized controlled trials and pilot studies further contributes to methodological inconsistency across studies.

As a result, this heterogeneity limits the direct comparability of findings and precludes the drawing of robust conclusions regarding the effectiveness of Perfetti’s method. It also restricts the ability to define a standardized intervention protocol in terms of frequency, intensity, and duration. Consequently, the current evidence should be interpreted as exploratory and hypothesis-generating rather than confirmatory.

In addition, given that the evidence base includes only one randomized controlled trial and several pilot studies, the observed effects may be overestimated. Therefore, the overall findings should be interpreted with caution, as they may not accurately reflect the true magnitude of the intervention effect.

Perfetti’s method has been widely applied in post-stroke rehabilitation since the 1970s; however, its effectiveness and safety have not yet been clearly established. Motor recovery after stroke is complex and multifactorial, and there is currently no consensus on the most effective neurorehabilitation approach. Although a wide variety of rehabilitation strategies have been investigated, no clear superiority over conventional occupational and physical therapy has been consistently demonstrated [41,42]. In this context, Perfetti’s method may offer a promising framework for integrating sensorimotor and cognitive processes in rehabilitation; however, its clinical superiority over conventional approaches remains uncertain. The integration of guided exercises within task-oriented movement may support motor learning through complex sensorimotor and cognitive interactions [6]. However, it is necessary to continue investigating the effectiveness of Perfetti’s method or the cognitive sensorimotor training therapy in the recovery of the upper-limb after a stroke, using high-quality methodological designs and standardized and recommended assessment tools. Future research should focus on a concrete neurological pathology and should describe the underpinning theoretical framework or the biological mechanisms intended to improve the recovery of outcomes, bearing in mind the factors concerning the patient that might impact the motor recovery (e.g., acute or chronic phase).

### Limitations

This systematic review has several limitations that should be considered. On the one hand, the sample sizes were very small and it was very difficult to draw robust conclusions from the studies. Moreover, only one RCT was included, as there is a lack of high-quality methodological studies. Therefore, the overall quality of the available evidence can be considered predominantly low. Consequently, the findings of this review should be regarded as exploratory and require confirmation through future high-quality studies. In this line, incomplete reporting of statistical outcomes in some of the included studies further limits the interpretation of the findings. In several cases, information regarding the statistical significance of within- or between-group differences was not reported (NR), making it difficult to fully assess the strength and consistency of the observed effects. This lack of reporting may introduce uncertainty and should be considered when interpreting the results of this review.

On the other hand, there was high heterogeneity among the included studies regarding the outcome variables and assessment tools, the protocol and application of the method, and the participants’ profile. In terms of comparisons of treatment effects, very few studies used designs based on intergroup comparisons, even though this is recommended in methodological reviews. Therefore, the results of the present systematic review should be interpreted with caution, particularly given the limited methodological quality of the included studies. Additionally, no search of grey literature or trial registries was conducted, which may have increased the risk of publication bias and the omission of relevant unpublished studies.

Although effect sizes were calculated to estimate the magnitude of change within individual studies, these values should be interpreted with caution. Due to the heterogeneity in study designs, populations, interventions, and outcome measures, effect sizes are not directly comparable across studies and do not represent pooled evidence. Therefore, they should be considered as descriptive indicators rather than definitive measures of comparative effectiveness.

Future research should include a larger sample size with a high-quality methodological design. Also, researchers should assess the upper limb’s functionality using the ARAT and body function using the FM scale, as is recommended by the Stroke Recovery and Rehabilitation Roundtable (SRRR) [41,42]. It is becoming urgent to adopt some recommendations in future research since it would significantly advance the quality, reproducibility, and rigor of stroke recovery and rehabilitation research, especially in those interventions that have shown no effectiveness since the intervention [41].

## 5. Conclusions

The present study results corroborate some clinical benefits of Perfetti’s method on stroke patients’ upper-limb function and dependence level. The included studies show some heterogeneity, making it difficult to draw robust conclusions regarding the effectiveness of the method. This review confirms the need for further studies on the effectiveness of Perfetti’s method in stroke patients’ upper-limb recovery.

## Data Availability

The data would be available upon request from the corresponding author.
